# A Comparative Study of Smartphone Game with Spirometry for Pulmonary Function Assessment in Stroke Patients

**DOI:** 10.1155/2018/2439312

**Published:** 2018-11-04

**Authors:** Sunghee Joo, Kyeongjin Lee, Changho Song

**Affiliations:** ^1^Department of Physical Therapy, College of Health Science, Sahmyook University, Republic of Korea; ^2^Department of Physical Therapy, College of Health Science, Kyungdong University, Republic of Korea

## Abstract

**Background:**

The monitoring and rehabilitation of pulmonary function can be immensely important for long-term performance of daily life activities in stroke patients. In recent times, smartphone game-based assessment (SGA) has been gaining in popularity as an alternative to laboratory assessments. Hence, the aims of this study were (1) to quantify the reliability and validity of SGA for pulmonary function and (2) to assess the validity of SGA in comparison to spirometry.

**Materials/Methods:**

Thirty-four stroke subjects (age = 49.24 ± 8.25 years) performed spirometry and the smartphone game on different days. Spirometric values were obtained using a spirometer (SP-1, Schiller, USA). A breathing game application (Breathing+ package, Breathing Labs, Slovenia) was used to obtain the values for the SGA of pulmonary function. The concurrent validity was determined by comparing data collected from the 2 systems, and the reliability was determined by comparing data collected from 3 sessions of using the breathing game on a smartphone.

**Results:**

All parameters demonstrated excellent agreement with intraclass correlation coefficients (ICC (2.1)) values for reliability and concurrent validity.

**Conclusion:**

We compared the relationship between the SGA and the spirometry as certified pulmonary function test. The SGA data were statistically significant and reliable for pulmonary function assessment in stroke patients. It will therefore be useful during rehabilitation to improve pulmonary function and clinical monitoring in stroke patients.

## 1. Background

Following a stroke, patients may have impairments such as loss of muscle mass, pain, and functional limitation. Reduced tolerance to daily physical activity can additionally lead to a more sedentary lifestyle which increases risk of pulmonary infections [1, 2]. Respiratory complications in stroke patients may occur due to changes in respiratory patterns or weakness of respiratory muscles. Respiratory complications such as decreased ventilation and cough effectiveness lead to difficulty in eliminating secretions, which significantly increases the risk of pulmonary diseases in stroke patients [3, 4]. In a detailed study of pulmonary function in stroke patients, researchers found that pulmonary function (maximum inspiratory and expiratory pressure) was significantly reduced. Therefore, it is important for stroke patients to achieve, improve, and maintain the physical capability of carrying out daily functions by monitoring and rehabilitation of pulmonary function [2, 5, 6].

Spirometry is widely used in clinical practice as it provides detailed and easy-to-interpret information on pulmonary function. The use of spirometry is necessary for the evaluation and follow-up of respiratory diseases and for confirmation of return to respiratory normality [7]. Spirometry is particularly essential for the monitoring and follow-up of respiratory diseases such as asthma and chronic obstructive pulmonary disease (COPD) and of the respiratory complications of stroke [8]. Thus, spirometry should be a part of any routine health examination of subjects at risk of developing pulmonary diseases [9]. However, there are limitations to the frequent use of spirometry in primary care [10]. Firstly, the spirometry is not a very portable apparatus, so it is not ideal for monitoring individual patients. Secondly, it is expensive as specific components consist of specialized disposable accessories [11]. Thirdly, its use requires special training and periodical refresher training of primary care professionals. [12, 13] Consequently, more convenient measures need to be developed and evaluated for use in clinical settings.

In recent times, smartphone game-based assessment (SGA) has been gaining in popularity as an alternative to laboratory assessments. These measurements are suggested as practical solutions for lowering cost as well as improving accessibility, convenience, and portability [14]. An SGA application requires that subjects perform physical activities and makes it possible for users to monitor their physical activity level while performing game-exercises [15]. Some studies have investigated the effectiveness of smartphone applications as assessment tools [14–16]. They found out that the use of the SGA can be suitable for clinician analysis. Breathing+ (Breathing+ package, Breathing Labs, Slovenia) is a SGA, downloadable on a laptop or smartphone and requiring a headset, which acts as a breathing trainer to enhance its user's respiratory function. Joo, Shin [17] reported enhanced respiratory function in stroke patients when intervention was conducted using this application. However, it is unknown whether this SGA can be used as an assessment tool beyond a single treatment protocol in the pulmonary rehabilitation of stroke patients. Hence, the aims of this study were (1) to quantify the reliability of SGA for pulmonary function and (2) to assess the validity of pulmonary function SGA in comparison to spirometry.

## 2. Materials and Methods

### 2.1. Subjects

Thirty-four stroke subjects (age = 49.24 ± 8.25 years) recruited from the 'B' rehabilitation hospital (Gyeonggi Province, South Korea), voluntarily participated in the study (20 males, 14 females; Height 165.16 ± 8.23 cm; Weight 61.19 ± 8.26 kg; BMI 22.41 ± 2.77 kg/m^2^). The inclusion criteria were a history of stroke of at least 6 months before the study; the ability to understand and follow simple verbal instructions; a Minimental State Examination-Korean version (MMSE-K) score >24 [18]; no facial palsy or unrestricted movement of the lips; a forced expiratory volume in one second (FEV_1_) < 93% of the predicted normal value [19]; and no history of orthopedic, neurologic, or cardiac conditions nor receptive aphasia, thoracic surgery or abdominal surgery. The exclusion criteria were a history of cardiac and/or chronic pulmonary disease; clinical signs of cardiac and/or pulmonary disease; presence of severe visual disability or visual field defects; inability to perform the tests; and the use of medications that could cause dizziness. All participants were provided verbal and written explanation of study procedures and they signed an informed consent form prior to participation. The University of Sahmyook Human Research Ethics Committee granted ethical approval.

### 2.2. Instruments

**Figure 1:**
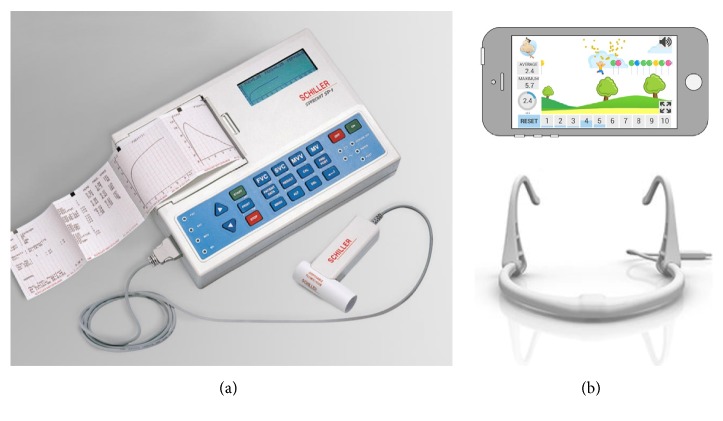
Two instrument systems: (a) spirometry (b) smartphone game application.

Spirometry is a physiological test that measures patient inspiratory and expiratory air volumes as functions of time. In this study, pulmonary function tests performed using the spirometer (SP-1, Schiller, USA) (Figure 1) and the SGA were compared. This spirometer unit satisfied all the American Thoracic Society (ATS, 2017) recommendations for spirometry [20]. Forced vital capacity (FVC), FEV_1_, predicted FVC (%), and predicted FEV_1_ (%) were the spirometric indices compared in this study because they are the most important spirometric parameters and the most commonly used indicators of disease severity in patients with pulmonary disease [21, 22]. FVC is the volume delivered during an expiration made as forcefully and as completely as possible after full inspiration, and FEV_1_ is the volume delivered in the first second of an FVC maneuver. [22]

Breathing+ was the game application used for the evaluation. (Figure 1) This game application consists of 14 different games, all designed to guide the participants to maximum inspiration and expiration through a headset. A smartphone was placed on the table, and the participant sat in a wheelchair or chair. An adjustable table was used to enable participants comfortably see the smartphone. Participants selected and played the games they were familiar with. The game result showed the longest exhalation period, and the average exhalation period in real time by measuring air pressure as detected through the headset. We converted and used the average real-time values for comparison.

### 2.3. Procedure

Two physiotherapists with experience of spirometry in pulmonary rehabilitation conducted the procedures. All participants satisfied the standard recommendations and quality criteria (acceptability and repeatability) of the American Thoracic Society/European Respiratory Society guidelines [20]. The patients first performed spirometry and then played the smartphone game (SG) two hours later. There was no physical therapy or exercise performed to minimize physiological changes [23]. The subjects were re-evaluated using the same procedure and instruments at the same time after 24 hours for test-retest reliability by the same therapist and again re-evaluated using the same procedure and instruments at the same time after 48 hours for interrater reliability by the other therapist in this study. This time interval was considered suitable to allow for stability of the instruments over time while minimizing biological variability [24]. Furthermore, feedback on test performance was provided only after conclusion of all procedures to minimize the risk of feedback from an earlier test influencing performance on the next test [23].

### 2.4. Statistical Analysis 

All measurements and values obtained from spirometry and SGA were evaluated for normality and homoscedasticity. Test-retest and interrater reliabilities for SGA were determined using an intraclass correlation coefficients (ICCs (2, 1)) with a 95% confidence interval (95% CI) [24]. The different parameters of the 2 systems were standardized for concurrent validity.

The coefficient of variation of method errors (CV_ME_) [25] and 95% limits of agreement (LOA) [26] were calculated for absolute comparison of values obtained from the 2 systems. In the formulas below, SD_d_ represents the standard deviation of the differences between the 2 tests and X_1_ and X_2_ represent the 2 tests means. CV_ME_ values were converted into percentages by calculating coefficients of variations of method errors collected using SD_d_.

(1) ME=SDd√2CVME=100×2MEX1+X2

In addition, the standard error of measurement (SEM) values was calculated using SD_d_ as the square root of the mean square error term from analysis of variance on data of test-retest and interrater reliabilities. It can alternatively be calculated using the formula below [27]:

(2) $$\begin{array}{c} SEM=SD\times \surd \left(\;1–ICC\;\right) \end{array}$$

And minimum detectable changes (MDC_95_) at a confidence level of 95%, also known as reliable change or smallest real difference, were calculated by multiplying the SEM by the* z*-score associated with the desired level of confidence and the square root of difference scores from measurements as below [28]:

(3) $$\begin{array}{c} {MDC}_{95}=\text{z-score }\left(\;\text{95\%CI}\;\right)\times \surd 2\times SEM \end{array}$$

All statistical calculations were completed using the MedCalc® 2011 statistical software (version 11.5.1).

## 3. Results

**Figure 2:**
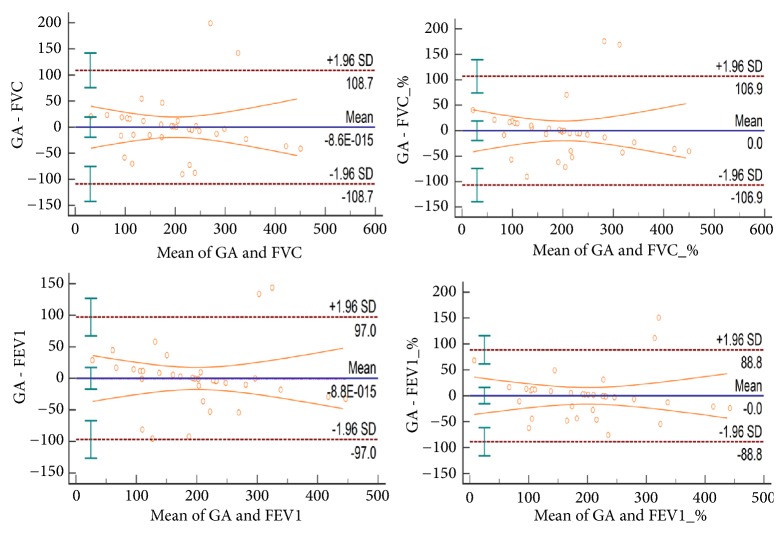
Bland-Altman Plot.

The mean and standard deviations of parameters of spirometry and SGA are presented in Table 1. ICC (2, 1) values for SGA parameters based on expiratory time are listed in Table 2. All parameters demonstrated excellent agreement with ICC (2.1) values at 0.84 (95%CI, 0.65-0.93) for intratest and 0.96 (95%CI, 0.91-0.98) for intertest. The SEM, SEM%, MDC, and MDC% for the SGA parameters are also listed in Table 2. The SEM% ranged from 0.41% to 4.41% while MDC% ranged from 1.16% to 12.22% and CV% ranged from 15.04% to 24.84%. These values indicate strong and absolute reliability and a low level of variation between the sessions (Table 2).  Table 3 shows ICC (2, 1) (95%CI), 95% LOA, and CV% values in correlation with each parameter between spirometry and SGA. The correlations are represented on the Bland Altman plot in Figure 2.

## 4. Discussion

Technological advances in the medical field have facilitated transition from bulky time-consuming devices to portable time-saving on such as smartphone that run application like Breathing+. The portable modern devices play an important, albeit indirect role in pulmonary function rehabilitation by measuring physiological effort. It was proposed that new methods based on recent technological advances must be evaluated and compared with established techniques. The purpose of this study was to evaluate SGA and compare it with spirometry. Overall, the results of this study suggest excellent reliability and acceptable measurement errors.

Relative reliability, which examines the relationship between repeated measurements and consistency among raters, can be evaluated using ICC [29]. According to Fleiss' classification, ICC values above 0.75 indicate excellent reliability, values between 0.40 and 0.75 indicate fair to good reliability, and values less than 0.40, indicate poor reliability [30]. In this study, ICC values showed a strong correlation between spirometry and SGA despite systematic differences (intra-test: 0.84, inter-test: 0.96) and are shown in Table 2. However, as suggested by Menz, Latt [31], a high ICC value does not necessarily mean excellent reliability even though ICC is a more appropriate indicator of reliability than simple correlation coefficients such as Pearson's or Spearman's rho. Therefore, both CV and LOA were calculated to decrease the intrinsic limitation effects and ensure excellent reliability. CV_ME_ represents the differences in values collected from the 2 systems as a percentage and can be used in clinical settings as an indicator of consistency since it is unaffected by sample heterogeneity [31]. The 95% LOA represents the expected range of difference between measurements, which is used to identify the presence of significant bias when they are repeatedly assessed. These results are shown in Tables 2 and 3. The SD_d_ of the test means that 0.92 and 0.18 were considered reliable. Bland and Altman [26] suggested the data not be used to assess repeatability if the SD_d_ differs significantly from zero.

SEM is calculated to estimate absolute reliability, which describes the within-participant variability attributable to repeated measures [29]. In this study, SEM calculated for intra- and intertests were expressed as percentages of the mean (SEM%) and showed a low level of measurement error, between 0.41% and -4.41%, which indicates strong absolute reliability. Small SEM values for SGA parameters indicate that SGA values were stable and reproducible over time, thereby implying precision in measurement [32]. A true change in the parameter of interest can be determined by assessing statistical significance and incorporating MDC or MDC% into clinical decision-making [29]. MDC indicates the minimum amount of change required to distinguish a true performance change from a change due to variability in performance or measurement error. [29] Since it is defined as the degree of sensitivity to change, MDC is used to determine actual occurrence of change over 2 measurement sessions [28]. Darter, Rodriguez [23] suggested that only MDC values less than 10% signify meaningful change. Relatively low MDC values (1.16%-12.22%) were obtained in this study when expressed as percentages of means. (Table 2) Moreover, as suggested by Bland and Altman [26], correlation analysis had to be followed by more specific investigations such as ICC and Bland-Altman plot to define agreement levels between the different systems. Figure 2 shows a correlation between parameters of spirometry and SGA. These findings indicate that SGA can confidently be used for clinical purposes as an alternative to spirometry as it circumvents the need for costly, frequent, and inconvenient testing in the clinic.

There are limitations to be recognized in this study. The sample size for our analysis was relatively small. It is possible that our results would have differed with a larger sample size. Although we have considered and standardized the real time SGA parameters with those of spirometry, the results of our study are not generalizable to other pulmonary function assessment devices. Another possible limiting factor of this study is the effect on results of learning during re-evaluation while using spirometry and/or SGA. The patients may have been motivated to use the instruments better through learning which would have affected the outcome.

This study is the first to investigate the reliability and validity of SGA in stroke patients. Conventional spirometers are not ideal for use in clinical settings for a variety of reasons, such as problems with continued use of damaged mouthpieces or incorrect use by patients. The SGA has the advantages of ease of setup, simplicity of use, and affordability which circumvent the shortcomings of conventional spirometers and make daily pulmonary function monitoring more attractive to patients. The SGA system could provide an interesting opportunity to promote home-based physical activity training sessions. Therefore, further research is needed to investigate differences in reliability and validity between the modern SGA system and the established spirometric setup in stroke patients with a view to optimizing outcome. In this study, excellent reliability and acceptable measurement errors were observed in SGA and spirometry. It is presumed that the study findings were due to the similarities between the SGA method requiring long-duration breathing and the spirometric measurement method for respiratory volumes and duration. This suggests that clinicians could benefit from using the SGA system by correlating SGA data to observed changes in functional status or to the quality of life postintervention.

## 5. Conclusion

We compared SGA and spirometry as certified pulmonary function test system. The SGA data were statistically significant and reliable for pulmonary function assessment in stroke patients. The SGA system will therefore be useful in clinical practice for the monitoring and rehabilitation of pulmonary function in stroke patients.

## Figures and Tables

**Table 1:** The mean and standard deviations of parameters of spirometry and SG.

Parameters		Mean ± SD
Spirometer	FVC (L)	3.26 ± 1.07
	FVC (%)	86.91 ± 25.92
	FEV_1_ (L)	2.47 ± 0.66
	FEV_1_ (%)	79.30 ± 18.36

GA	Real-time (s)	8.5 ± 0.69

FVC=forced vital capacity; FEV_1_=forced expiratory volume in 1 second; GA=game-based assessment; SG=smartphone game.

**Table 2:** Mean ± SD and reliability measured with smartphone-game application.

	Mean ± SD	ICC (2,1) 95%CI	95% LOA	SEM	SEM (%)	MDC	MDC (%)	CV (%)
Intra-test	8.4 ± 0.92	0.84	0.30~2.33	0.37	4.41	1.02	12.22	24.84
		(0.65-0.93)						

Inter-test	8.9 ± 0.18	0.96	1.66~3.80	0.3	0.41	0.10	1.16	15.04
		(0.91-0.98)						

CV=coefficients of variation of method error; ICC=intracorrelation coefficient; LOA=limits of agreement; MDC=minimum detectable change; SEM=standard error of measurement.

**Table 3:** Concurrent validity measured with spirometry and smartphone-game application.

Parameters	ICC (2,1) 95%CI	95% LOA	CV ( %)
FVC - GA	0.91 (0.83-0.95)	75.35~142.22	19.34

FVC % - GA	0.92 (0.83-0.96)	74.22~140.03	19.03

FEV_1_ – GA	0.93 (0.86-0.96)	67.12~126.72	17.23

FEV_1_ % - GA	0.94 (0.89-0.97)	61.67~116.37	15.82

CV=coefficients of variation of method error; FVC=forced vital capacity; FEV_1_=forced expiratory volume in 1 second; GA=game-based assessment; SG=smartphone game; ICC=intracorrelation coefficient; LOA=limits of agreement.

## Data Availability

The Excel data used to support the findings of this study are included within the article.

## References

[1] Eng J. J., Dawson A. S., Chu K. S. (2004). Submaximal exercise in persons with stroke: test-retest reliability and concurrent validity with maximal oxygen consumption. *Archives of Physical Medicine and Rehabilitation*.

[2] Luvizutto G. J., dos Santos M. R. L., Sartor L. C. A. (2017). Evaluation of Respiratory Muscle Strength in the Acute Phase of Stroke: The Role of Aging and Anthropometric Variables. *Journal of Stroke and Cerebrovascular Diseases*.

[3] Zhou Z., Vincent F., Salle J.-Y., Antonini M.-T., Aliamus V., Daviet J.-C. (2012). Acute stroke phase voluntary cough and correlation with maximum phonation time. *American Journal of Physical Medicine & Rehabilitation*.

[4] Yoon J., Park J., Lee D., Roh H. (2012). Comparisons of respiratory function and activities of daily living between spinal cord injury and stroke patients and normal elderly people. *Journal of Physical Therapy Science*.

[5] Harraf F., Ward K., Man W. (2008). Transcranial magnetic stimulation study of expiratory muscle weakness in acute ischemic stroke. *Neurology*.

[6] Ward K., Seymour J., Steier J. (2010). Acute ischaemic hemispheric stroke is associated with impairment of reflex in addition to voluntary cough. *European Respiratory Journal*.

[7] García-Río F., Calle M., Burgos F. (2013). Espirometría. *Archivos de Bronconeumología*.

[8] Peña V. S., Miravitlles M., Gabriel R. (2000). Geographic variations in prevalence and underdiagnosis of COPD: Results of the IBERPOC multicentre epidemiological study. *CHEST*.

[9] García-Río F., Calle M., Burgos F. (2013). Spirometry. *Archivos de Bronconeumología (English Edition)*.

[10] Caramori G., Bettoncelli G., Tosatto R. (2005). Underuse of spirometry by general practitioners for the diagnosis of COPD in Italy. *Monaldi Archives for Chest Disease - Pulmonary Series*.

[11] Pauwels R. A., Buist A. S., Calverley P. M. A., Jenkins C. R., Hurd S. S. (2001). Global strategy for the diagnosis, management, and prevention of chronic obstructive pulmonary disease: NHLBI/WHO Global Initiative for Chronic Obstructive Lung Disease (GOLD) workshop summary. *American Journal of Respiratory and Critical Care Medicine*.

[12] Bellia V., Pistelli R., Catalano F. (2000). Quality control of spirometry in the elderly: The SA.R.A. study. *American Journal of Respiratory and Critical Care Medicine*.

[13] Schermer T. R., Jacobs J. E., Chavannes N. H. (2003). Validity of spirometric testing in a general practice population of patients with chronic obstructive pulmonary disease (COPD). *Thorax*.

[14] Silsupadol P., Teja K., Lugade V. (2017). Reliability and validity of a smartphone-based assessment of gait parameters across walking speed and smartphone locations: Body, bag, belt, hand, and pocket. *Gait & Posture*.

[15] Sgrò F., Monteleone G., Pavone M., Lipoma M. (2014). Validity Analysis of Wii Balance Board Versus Baropodometer Platform Using an Open Custom Integrated Application. *AASRI Procedia*.

[16] Yamada M., Aoyama T., Nakamura M. (2011). The Reliability and Preliminary Validity of Game-Based Fall Risk Assessment in Community-Dwelling Older Adults. *Geriatric Nursing*.

[17] Joo S., Shin D., Song C. (2015). The effects of game-based breathing exercise on pulmonary function in stroke patients: A preliminary study. *Medical Science Monitor*.

[18] Polese J. C., Ada L., Teixeira-Salmela L. F. (2018). Relationship between oxygen cost of walking and level of walking disability after stroke: An experimental study. *Physiotherapy Research International*.

[19] Barreiro E., Coronell C., Laviña B., Ramírez-Sarmiento A., Orozco-Levi M., Gea J. (2006). Aging, sex differences, and oxidative stress in human respiratory and limb muscles. *Free Radical Biology & Medicine*.

[20] Chushkin M., Popova L., Ots O., Nenasheva O., Struchkov P. (2017). Comparison between results of spirometry performed with the ATS/ERS quantitative criteria and simple visual inspection criteria. *European Respiratory Journal*.

[21] Leidy N. K. (1995). Functional performance in people with chronic obstructive pulmonary disease. *Image: the Journal of Nursing Scholarship*.

[22] Miller M. R., Hankinson J., Brusasco V. (2005). Standardisation of spirometry. *European Respiratory Journal*.

[23] Darter B. J., Rodriguez K. M., Wilken J. M. (2013). Test-retest reliability and minimum detectable change using the K4b 2: Oxygen consumption, gait efficiency, and heart rate for healthy adults during submaximal walking. *Research Quarterly for Exercise and Sport*.

[24] Fong K. N., Ng B. H., Chow K. K. (2001). Reliability and Validity of the Monitored Functional Task Evaluation (MFTE) for Patients with Chronic Obstructive Pulmonary Disease (COPD). *Hong Kong Journal of Occupational Therapy*.

[25] Portney L. G., Watkins M. P. (2000). *Foundations of Clinical Research: Applications to Practice*.

[26] Martin Bland J., Altman D. (1986). Statistical methods for assessing agreement between two methods of clinical measurement. *The Lancet*.

[27] Fleiss J. L. (2011). *Design And Analysis of Clinical Experiments*.

[28] Haley S. M., Fragala-Pinkham M. A. (2006). Interpreting change scores of tests and measures used in physical therapy. *Physical Therapy in Sport*.

[29] Mohandas Nair P., George Hornby T., Louis Behrman A. (2012). Minimal detectable change for spatial and temporal measurements of gait after incomplete spinal cord injury. *Topics in Spinal Cord Injury Rehabilitation*.

[30] Fleiss J. L. (1972). Classification of the depressive disorders by numerical typology. *Journal of Psychiatric Research*.

[31] Menz H. B., Latt M. D., Tiedemann A., Kwan M. M. S., Lord S. R. (2004). Reliability of the GAITRite® walkway system for the quantification of temporo-spatial parameters of gait in young and older people. *Gait & Posture*.

[32] Beckerman H., Roebroeck M. E., Lankhorst G. J., Becher J. G., Bezemer P. D., Verbeek A. L. M. (2001). Smallest real difference, a link between reproducibility and responsiveness. *Quality of Life Research*.

